# Association between tumor necrosis factor polymorphisms and rheumatoid arthritis as well as systemic lupus erythematosus: a meta-analysis

**DOI:** 10.1590/1414-431X20187927

**Published:** 2019-03-25

**Authors:** Lin Chen, Zhuochun Huang, Yun Liao, Bin Yang, Junlong Zhang

**Affiliations:** 1 Key Laboratory of Birth Defects and Related Diseases of Women and Children, Ministry of Education, Department of Obstetrics and Gynecology, West China Second University Hospital, Sichuan University, Chengdu, China; 2 Department of Laboratory Medicine, West China Hospital, Sichuan University, Chengdu, China

**Keywords:** Rheumatoid arthritis, Single-nucleotide polymorphism, Systemic lupus erythematosus, Tumor necrosis factor-alpha, Meta-analysis

## Abstract

Tumor necrosis factor-alpha (*TNF-α*) plays an important role in autoimmune diseases. Previous studies have investigated the association of *TNF-α-238G/A* (rs361525) and *-308G/A* (rs1800629) polymorphisms with rheumatoid arthritis (RA) and systemic lupus erythematosus (SLE). However, no agreed conclusion had been made. Therefore, this meta-analysis was conducted to assess the associations of *TNF-α-238G/A* and *-308G/A* polymorphisms with RA and SLE risk. A systematic search was conducted in commonly used databases. Meta-analysis was performed by STATA12.0. A total of 43 studies were included. In the overall population, the *TNF-α-238A* allele was observed to be a protective factor for RA (A *vs* G: OR=0.75, 95%CI=0.57–0.99, P=0.040) and the *TNF-α-308A* allele was found to be a risk factor for SLE (A *vs* G: OR=1.78, 95%CI=1.45–2.19, P<0.001). However, no evidence of association was found between *TNF-α*-238 G/A polymorphism and SLE nor between -308G/A and RA. In the subgroup analysis, *TNF-α-308A* allele played a pathogenic role for RA in Latin Americans (A *vs* G: OR=1.46, 95%CI=1.15–1.84, P=0.002) and for SLE in Latin Americans (A *vs* G: OR=2.12, 95%CI=1.32–3.41, P=0.002) and Europeans (A *vs* G: OR=2.03, 95%CI=1.56–2.63, P<0.001), while it played a protective role for RA in Asians (A *vs* G: OR=0.54, 95%CI=0.32–0.90, P=0.017). No significant association was found between *TNF-α-308G/A* and SLE susceptibility in Africans and Asians. This meta-analysis demonstrated that *TNF-α-238A* was associated with decreased risk of RA rather than SLE, while *-308G/A* polymorphism was associated with SLE rather than RA. Stratification analysis indicated that different ethnicities would have different risk alleles.

## Introduction

Rheumatoid arthritis (RA) and systemic lupus erythematosus (SLE) are both chronic systemic autoimmune diseases. RA is associated with systemic and chronic inflammation of the joints, resulting in synovitis and pannus formation. It is a serious pathological condition that can lead to incapacitation and decreased life expectancy compared with the normal population (1). People suffering from RA accounts for around 1% of the world's population (2). Simultaneously, SLE is characterized by the production of multiple autoantibodies, complement activation, and immune-complex deposition, resulting in tissue and organ damage (3). Compared with the general population, the risk of death in patients with SLE increases threefold (4). RA and SLE cause serious threats to human health, while their etiology and pathogenesis are still unclear (5).

For a long period, given the importance of cytokines in immune system regulation, several circulating cytokines, especially tumor necrosis factor-alpha (TNF-α), abnormalities have been reported in RA and SLE (6,7). TNF-α is a potent pro-inflammatory cytokine that stimulates cytokine production, enhances expression of adhesion molecules, increases neutrophil activation, and acts as a costimulator for Tcell activation and antibody production (8). It plays an important role in inflammatory and immune responses. TNF-α is coded and regulated by *TNF-α* gene, which is located in chromosome 6, within the class III region of MHC (9). Several studies analyzed the association of *TNF-α* gene with susceptibility to RA and SLE (10–12) and numbers of single-nucleotide polymorphisms (SNPs) of *TNF-α* gene were identified. Among these, two common polymorphisms in the promoter, G to A substitution at position -238 (*TNF-α-238G/A,* rs361525) and position -308 (*TNF-α-308G/A,* rs1800629), attracted widespread attention. Several case-control studies were conducted to investigate the association of *TNF-α-238G/A* and *-308G/A* with RA and SLE. However, significant associations with these two polymorphisms related to RA and SLE were not consistently observed. Some studies indicated a significant association (13–15) while others suggested not (16–18). The inconsistency could be the result of limited sample size of some of the studies, ethnicity, etc. Meta-analyses were performed to illuminate the controversy (19,20). These meta-analyses demonstrated that the *TNF-α-308 G/A* polymorphism was associated with susceptibility to SLE and might represent a significant risk factor for RA in Latin Americans but not in Europeans. However, the number of studies included in these meta-analyses was small and some of the studies were not available for Hardy-Weinberg equilibrium. In recent years, more studies focused on the association of *TNF-α-308G/A* with RA/SLE in Asians (13,16,17,21) and the association of another SNP *TNF-α-238G/A* with risk of RA/SLE (22,23). It would be helpful to discern the contribution of each SNP in the *TNF-α* gene to the risk of RA/SLE through analyzing their effects. It is imperative to perform a meta-analysis to evaluate the association between the two SNPs and RA/SLE risk. Therefore, this meta-analysis was conducted with published data to investigate whether the *TNF-α* promoter-238 G/A and -308 G/A polymorphisms contribute to the susceptibility to RA and SLE.

## Material and Methods

### Search strategy

A comprehensive search was conducted until July 31, 2018 in Medline, Embase, Chinese Biomedical Literature Database (CBM), and China National Knowledge Infrastructure (CNKI). “Tumor necrosis factor-alpha”, “*TNF-α*”, “polymorphism”, “variant”, “rheumatoid arthritis”, “RA”, “systemic lupus erythematosus”, and “SLE” were searched as both medical subject heading (MeSH) terms and text words. In addition, the reference citations in the obtained articles were scrutinized to guarantee not missing eligible studies.

### Criteria for article screening

For inclusion, a study had to: *a*) be a case-control study and based on original data (independence among studies); *b*) assess the presence of the variant *TNF-α-238G/A* and/or *TNF-α-308G/A* in the *TNF-α* gene of patients with and without RA or SLE; *c*) provide sufficient data to calculate odds ratio (OR). We excluded the following: *a*) no control group; *b*) studies containing overlapping data (if two inclusion times overlapped for more than 30% of the study time or all the patients were from the same region, only the latest article or the one with the larger case number was adopted); *c*) no certain number of the null and/or wild genotypes.

### Data extraction

The following information was extracted from the studies: first author's name, publication time, country, ethnicity, study design, sequencing method, number of cases (RA or SLE patients) and controls (healthy donors), mean age, female proportion, genotype distribution, and mutation sites. All data were collected by two members independently. The other investigators were consulted to reach a consensus when any divergence occurred.

### Quality assessment

The quality assessment of the included studies were conducted by two members independently according to the quality assessment scale (24). In this scale, five items were carefully checked, which included the representativeness of cases, source of controls, sample size, quality control of genotyping methods, and Hardy-Weinberg equilibrium. The quality score ranges from 0 to 10 and higher scores means better quality of the study. Two investigators scored the studies independently and solved disagreement through discussion.

### Statistical analysis

The meta-analysis was conducted based on the PRISMA checklists and the guidelines (25). Hardy-Weinberg equilibrium (HWE) was evaluated for each study by the chi-squared test in control groups and P<0.05 was considered a significant departure from HWE. Deviation from HWE among controls could imply some potential biases in the selection of control or genotyping errors, so only the studies without deviation from HWE among controls were used to do a subsequent meta-analysis. The impact of the polymorphisms on RA/SLE was estimated by summary OR and their corresponding 95%CI. Pooled ORs were performed for heterozygote model (GA *vs* GG), dominant model (GA+AA *vs* GG), and allelic comparison (A *vs* G). The overall effect was appraised through the Z test, which could be deemed significant if the P value was less than 0.05. The heterogeneity for the included articles was evaluated with Cochran's Q test, I^2^ statistics (the heterogeneity could be accepted if P>0.1 and I^2^ ≤50%) (26). If the value of I^2^ statistics was less than 50% or the P value was greater than 0.1, the fixed-effects model can be used, otherwise, random-effects model would be used. Begg's funnel plot and Egger's test were performed to examine publication bias (27). Besides, subgroup analyses were done by ethnicity. In addition, analysis was conducted after stratifying by the quality score (low quality group: score <6, high quality group: score ≥6) to make the results more credible. Statistical analyses were performed by STATA version 12.0 (USA). All tests were two-sided.

## Results

### Studies included in the meta-analysis

**Figure 1:**
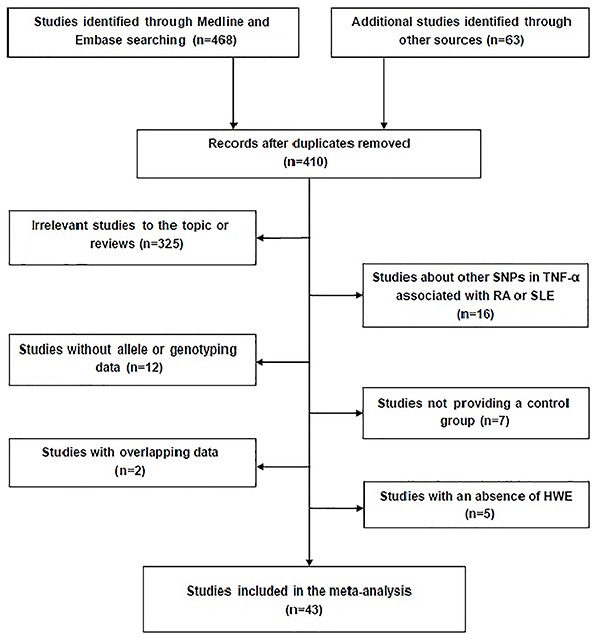
Flow chart illustrating the selection of articles included in the meta-analysis. SNP: single-nucleotide polymorphisms; RA: rheumatoid arthritis; SLE: systemic lupus erythematosus; HWE: Hardy-Weinberg equilibrium.

The flow chart (Figure 1) describing the screening process was modified according to the PRISMA Statement (25). A total of 531 studies were acquired from databases. After skimming the titles and abstracts, 446 articles were excluded, of which 121 articles were duplicates and 325 articles were not related to this topic. The remaining 85 studies were included for full-text review, and 37 studies were excluded, among which, 16 articles were with other SNPs in *TNF-α* and 12 articles were without allele or genotyping data, 7 articles were not case-control studies, 2 articles were with overlapping data (reference not shown). Another 5 studies were excluded due to the absence of Hardy-Weinberg equilibrium (reference not shown). Finally, a total number of 43 relevant studies that met the inclusion criteria were included in this meta-analysis, among which, 14 case-control studies focused on *TNF-α-238G/A* and 42 studies focused on *TNF-α-308G/A*.

In the studies whose genotype frequencies of *TNF-α-238G/A* and *TNF-α-308G/A* were presented separately, each of them was treated as separate studies. In the end, in terms of *TNF-α-238G/A*, 8 studies containing 1386 cases and 1535 controls for RA and 7 studies involving 1296 cases and 1558 controls for SLE were included in this meta-analysis. In terms of *TNF-α-308G/A*, 19 studies including 3503 cases and 3993 controls for RA and 26 studies involving 3051 cases and 4232 controls for SLE were included in this meta-analysis.

### Association between *TNF-* **α**-*238G/A* polymorphism and RA/SLE susceptibility

**Figure 2:**
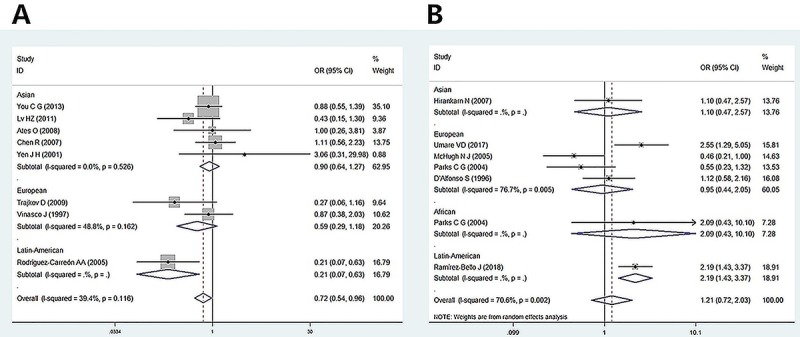
Forest plot for the correlation between *TNF-α*-238 and rheumatoid arthritis **(A)** and systemic lupus erythematosus **(B)** risk.

The summary of the meta-analysis for the association of *TNF-α-238G/A* polymorphism with RA is shown in Table 1. No significant heterogeneity was identified by the Q-test and I^2^ statistic except in European allelic comparison. Therefore, fixed-effects model was used except for the European study. Overall, an association was found in every genetic model. However, stratification by ethnicity indicated that *TNF-α-238G/A* was not significantly associated with RA in Asians, while an association was found in Latin Americans (Figure 2A).

**Table 1:** Summary of pooled odds ratio (OR) and 95% confidence interval (CI) for the association of *TNF-α*-238 single-nucleotide polymorphisms with rheumatoid arthritis (RA) and systemic lupus erythematosus (SLE).

*TNF-α*-238	Population	No. of studies	OR (95%CI)	P_OR_	I^2^	Effect model	P_Egger_
RA							
GA *vs* GG	Overall	8	0.71 (0.53-0.95)	0.020	46.00%	F	0.419
	Asian	5	0.89 (0.63–1.26)	0.514	0.00%	F	
	European	2	0.61 (0.30–1.23)	0.168	40.00%	F	
	Latin American	1	0.11 (0.03–0.48)	0.003		F	
GA+AA *vs* GG	Overall	8	0.72 (0.54–0.96)	0.026	39.40%	F	0.497
	Asian	5	0.90 (0.64–1.27)	0.549	0.00%	F	
	European	2	0.59 (0.29–1.18)	0.137	48.80%	F	
	Latin American	1	0.21 (0.07–0.63)	0.005		F	
A *vs* G	Overall	8	0.75 (0.57–0.99)	0.040	28.60%	F	0.515
	Asian	5	0.92 (0.66–1.27)	0.599	0.00%	F	
	European	2	0.55 (0.16–1.85)	0.334	55.90%	R	
	Latin American	1	0.32 (0.13–0.81)	0.015		F	
SLE							
GA *vs* GG	Overall	7	1.10 (0.60–2.01)	0.756	75.60%	R	0.205
	European	4	0.79 (0.32–1.96)	0.615	80.20%	R	
	Asian	1	1.10 (0.47–2.57)	0.828		R	
	African	1	2.09 (0.43–10.10)	0.360		R	
	Latin American	1	2.25 (1.45–3.49)	0.000		R	
GA+AA *vs* GG	Overall	7	1.21 (0.72–2.03)	0.481	70.60%	R	0.298
	European	4	0.95 (0.44–2.05)	0.892	76.70%	R	
	Asian	1	1.10 (0.47–2.57)	0.828		R	
	African	1	2.09 (0.43–10.10)	0.360		R	
	Latin American	1	2.19 (1.43–3.37)	0.000		R	
A *vs* G	Overall	7	1.30 (0.84–2.00)	0.238	62.10%	R	0.359
	European	4	1.09 (0.57–2.10)	0.786	72.30%	R	
	Asian	1	1.10 (0.48–2.52)	0.832		R	
	African	1	2.06 (0.43–9.81)	0.366		R	
	Latin-American	1	2.05 (1.36–3.09)	0.001		R	

R: random effect; F: fixed effect; P_Egger_: P value of Egger’s test.

The correlation between the *TNF-α-238G/A* polymorphism and SLE were also evaluated. Seven studies consisting of four European, one Asian, one African, and one Latin American were included in the meta-analysis and significant statistical heterogeneity was observed, thus random-effects model was used. No association between SLE and *TNF-α-238G/A* was found in the overall population. Similarly, subgroup analysis was conducted according to ethnicity. No significant association was found in Europeans in any of the genetic models. However, an association was found in Latin Americans (see Table 1; Figure 2B).

Figures 2A and B presents the OR and 95%CI for individual studies and pooled data for the association between *TNF-α*-238 and RA and between *TNF-α*-238 and SLE in the dominant model, respectively.

### Association between *TNF*-**α**-*308G/A* polymorphism and RA/SLE susceptibility

Forty-two articles including 3503 RA cases, 3051 SLE cases, and 8225 controls were included for *TNF-α-308 G/A*.

**Figure 3:**
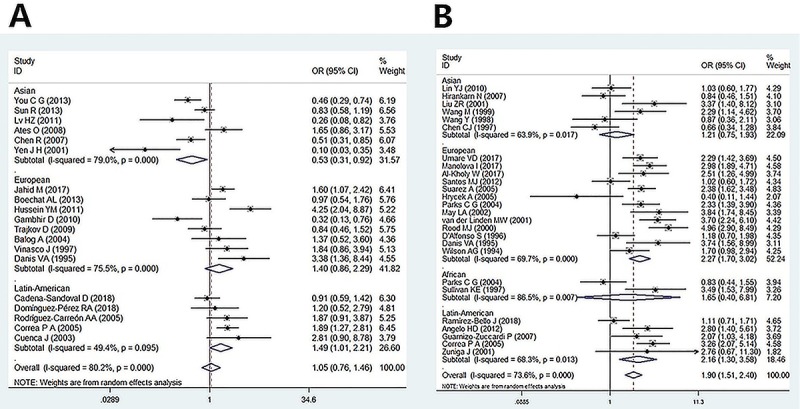
Forest plot for the correlation between *TNF-α*-308 and rheumatoid arthritis **(A)** and systemic lupus erythematosus **(B)** risk.

In the analysis of the association between *TNF-α-308G/A* polymorphism and the susceptibility to RA, significant heterogeneity was always observed except in Latin Americans. Thus, the random-effects model was used for overall, Asian and European, while the fixed-effects model was used for Latin American. Overall, no association was found in any of the genetic models. In subgroup analysis by ethnicity, *TNF-α-308G/A* was found to be associated with the susceptibility to RA in Asians and Latin Americans. However, no association was found to TNF–a*–308G/A* with RA patients in Europeans (see Table 2; Figure 3A).

**Table 2:** Summary of pooled odds ratio (OR) and 95% confidence interval (CI) for the association of *TNF-α*-308 single-nucleotide polymorphisms with rheumatoid arthritis (RA) and systemic lupus erythematosus (SLE).

*TNF-α*-308	Population	No. of studies	OR (95%CI)	P_OR_	I^2^	Effect model	P_Egger_
RA							
GA *vs* GG	Overall	19	1.02 (0.75–1.40)	0.878	77.70%	R	0.823
	Asian	6	0.54 (0.31–0.95)	0.031	79.30%	R	
	European	8	1.32 (0.82–2.13)	0.249	72.40%	R	
	Latin American	5	1.40 (1.08–1.81)	0.011	44.50%	F	
GA+AA *vs* GG	Overall	19	1.05 (0.76–1.46)	0.748	80.20%	R	0.890
	Asian	6	0.53 (0.31–0.92)	0.023	79.00%	R	
	European	8	1.40 (0.86–2.29)	0.179	75.50%	R	
	Latin American	5	1.45 (1.13–1.87)	0.004	49.40%	F	
A *vs* G	Overall	19	1.06 (0.78–1.45)	0.693	81.20%	R	0.761
	Asian	6	0.54 (0.32–0.90)	0.017	77.50%	R	
	European	8	1.40 (0.89–2.20)	0.145	76.60%	R	
	Latin American	5	1.46 (1.15–1.84)	0.002	48.70%	F	
SLE							
GA *vs* GG	Overall	26	1.81 (1.46–2.26)	0.000	68.50%	R	0.557
	Asian	6	1.17 (0.76–1.81)	0.480	55.80%	R	
	European	13	2.18 (1.69–2.82)	0.000	59.50%	R	
	African	2	1.64 (0.33–8.15)	0.543	88.60%	R	
	Latin American	5	1.94 (1.20–3.13)	0.007	61.90%	R	
GA+AA *vs* GG	Overall	26	1.90 (1.51–2.40)	0.000	73.60%	R	0.678
	Asian	6	1.21 (0.75–1.93)	0.434	63.90%	R	
	European	13	2.27 (1.70–3.02)	0.000	69.70%	R	
	African	2	1.65 (0.40–6.81)	0.486	86.50%	R	
	Latin American	5	2.16 (1.30–3.58)	0.003	68.30%	R	
A *vs* G	Overall	26	1.78 (1.45–2.19)	0.000	74.30%	R	0.522
	Asian	6	1.19 (0.77–1.85)	0.437	66.50%	R	
	European	13	2.03 (1.56–2.63)	0.000	80.10%	R	
	African	2	1.55 (0.54–4.42)	0.413	80.10%	R	
	Latin American	5	2.12 (1.32–3.41)	0.002	70.20%	R	

R: random effect; F: fixed effect; P_Egger_: P value of Egger’s test.

In terms of *TNF-α-308G/A* polymorphism and association with SLE, significant heterogeneity was identified and random-effects model was employed to evaluate the association. A significantly increased risk of SLE was observed in all comparisons in overall population. In subgroup analysis by ethnicity, *TNF-α-308G/A* strongly correlated with the risk of SLE in Europeans and Latin Americans even after adjustment for heterogeneity, while no such association was observed in Asians and Africans (see Table 2; Figure 3B).

Figure 3A and B presents the OD and 95%CI for individual studies and pooled data for the association between *TNF-α*-308 and RA and between *TNF-α*-308 and SLE in the dominant model, respectively.

### Evaluation of publication bias

**Figure 4:**
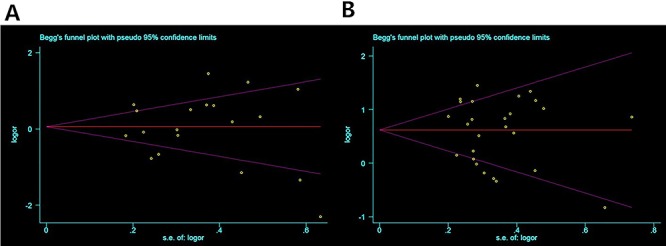
Funnel plots of publication bias for meta-analysis of *TNF-α*-308 and rheumatoid arthritis **(A)** and systemic lupus erythematosus **(B)**.

Publication bias was evaluated by Egger's test and Begg's funnel plot. The Egger's test P value was greater than 0.1 in all comparisons, showing that there was no evidence of publication bias in this meta-analysis (See Tables 1 and 2; Figure 4).

Figure 4A and B presents the funnel plot for publication bias for *TNF-α-308* polymorphism and RA and for *TNF-α-308* polymorphism and SLE on allelic contrast overall.

### Sensitivity analysis

Sensitivity analysis was performed to examine the influence set by sequential omission of every study in every genetic model. The odds ratio was not significantly influenced by omitting any single study for every genetic mode.

## Discussion

The *TNF-α* gene encodes the TNF-α cytokine, a multifunctional protein and one of the major regulators of inflammation that is involved in different normal immunological processes (8,28). Moreover, TNF-α cytokine plays an important role in pathological processes. For example, it induces its own secretion in macrophages, orchestrates tissue recruitment of immune cells, promotes tissue destruction, and stimulates the synthesis of inflammatory cytokines, chemokines, and different cell survival factors (8,28,29). Additionally, *TNF-α* is related to acute/chronic inflammation and has been found to be associated with several inflammatory and autoimmune diseases (18,28–30). The *TNF-α* gene promoter contains several SNPs, including two SNPs located at the positions -308G/A (rs1800629) and -238G/A (rs361525) of transcription start site, respectively (28). In addition, many studies have focused on the association between *TNF-α* gene polymorphisms and RA/SLE, but no consistent results had been made. To produce a more convincing conclusion, we conducted this study. It is the first meta-analysis to assess the association between *TNF-α-238G/A* and RA.

Our study showed that *TNF-α-238A* allele was associated with RA as a protective factor but not related to SLE susceptibility in the overall population. In subgroup analysis by ethnicity in the association analysis of *TNF-α*-238G/A and RA, no association was found except one study that showed an association between *TNF-α-238G/A* and RA susceptibility in Latin Americans. In the association analysis of *TNF-α*-238G/A and SLE, another study showed that *TNF-α-238A* allele correlated with SLE as a risk factor. However, these findings should be interpreted with caution due to the limited number of studies. Nevertheless, it is worth noting that in the stratification analysis by quality score, the association was found between *TNF-α-238G/A* and SLE in Europeans in the high quality group. As only seven studies were included in the analysis, it may not have enough power to support the association between them. More Latin American studies about the correlation between *TNF-α*-238 and SLE should be carried out to clarify this possible association. At the same time, our results indicated that *TNF-α-308G/A* polymorphism was associated with SLE. By subgroup analysis, a significantly increased risk was observed in patients with *TNF-α-308A* allele in Latin Americans and Europeans, while no significant association was found in Asians and Africans. Besides, no correlation was found between *TNF-α-308G/A* and RA in the overall population. But in subgroup analysis, it was found that *TNF-α-308A* played a protective role for RA in Asians, while a pathogenic role for RA was found in Latin Americans.

In short, the meta-analysis showed a possible role of ethnic differences in genetic backgrounds. In addition, the differences can be explained by the different life styles, genetic heterogeneity, etc. The influence of the *TNF-α-238A* or *-308A* allele might be masked by the presence of other as-yet unidentified reasons involved in RA or SLE development.

The role of *TNF-α* in autoimmunity may vary in different diseases. Compelling evidence indicated a pathogenic role of this cytokine in RA (31), while a protective role has been found in SLE (32). Our results indicated that the *TNF-α* gene polymorphisms constituted a common susceptibility factor for RA and SLE. These findings sustained the common disease hypothesis, which emphasizes that many disease genes may not be disease-specific, and that similar immunogenetic mechanisms underlie these diseases (33,34). Besides, our meta-analysis showed ethnicity may influence the role of the *TNF-α* gene in disease susceptibility. The question is how to relate these genetic findings with cytokine function in disease.

The premise of this meta-analysis of *TNF-α* polymorphisms was that gene variants with a significant pathological role would lead to a greater understanding of the regulatory mechanisms in both health and disease, and may provide more knowledge for identifying and allowing early intervention in at-risk individuals (35). Meanwhile, some studies have associated SNP of the *TNF-α* gene with cytokine synthesis and evidence suggests that there was an effect of the *TNF-α-238G/A* and *-308G/A* polymorphisms on *TNF-α* transcription (36,37). *TNF-α-308A* was a much stronger transcriptional activator than the common allele (G) in human B cell line, which may be related with the generation of a hypersensitive site at position -308 and an adjacent area of protection (38). However, the function of *TNF-α-238A* in *TNF-α* transcription was uncertain since some studies indicated that A allele or GA genotype affected gene expression while other studies did not (39,40). TNF-α cytokine has several functions such as stimulating the generation of inflammatory cytokines, promoting neutrophil activation and expression of adhesion molecules, and performing as a costimulator for T cell activation and antibody production. Overproduction of TNF-α might play an important role in susceptibility to the development of autoimmunity. Therefore, genetic variants of *TNF-a* may have an effect on the susceptibility to autoimmune disease development and on its clinical manifestations.

Several strengths characterize this meta-analysis. Firstly, it is the first meta-analysis that focused on the association between two SNPs of the *TNF-α* gene and the susceptibility to RA and SLE. In addition, compared with the former meta-analysis about the association between *TNF-α*-308 and RA (19), more studies especially about Asians were included and supplementary analyses including subgroup and sensitivity analyses were performed. Moreover, to minimize the risk of publication bias, the combined searches were conducted from a number of databases as well as all abstracts presented in English at RA and SLE congresses over the last 2 years and were extensively screened. Statistical methods were also used to test the publication bias, and no publication bias was identified by either Begg's funnel plot or Egger's regression test. Finally, we scored every study and then conducted the meta-analysis again after excluding the studies that got low scores, which made the results more credible.

However, this study has several potential limitations. Firstly, a large amount of studies was included in this meta-analysis and inevitably resulted in heterogeneity, which could not be eliminated easily. Random-effects model was selected to reduce the potential bias. Additionally, the sensitivity analysis was conducted and the ORs were not significantly affected. Therefore, the influence on the accuracy of our results was limited. Secondly, when taking subgroup analysis, a small number of studies were eligible and the evidence seemed to be insufficient and unconvincing. Finally, only the data of articles published in English or Chinese were extracted and a potential bias might thus have been introduced. The findings in this study should be interpreted prudently considering these limitations.

The results of stratification analysis still need more large-scale, well-designed case-control studies to be proven. However, our study provided an argument for performing further mechanistic studies to better understand the role of the *TNF-α* gene in RA or SLE development. Further evaluation of the effect of gene-gene and gene-environment interactions on the *TNF-α* promoter-238G/A and *-308G/A* polymorphism and RA/SLE susceptibility is necessary. Moreover, in terms of variants in *TNF-α* at position -238 or -308, whether and how this change promotes the inflammation process of RA or SLE requires further investigation.

## References

[1] Gabriel SE, Crowson CS, Kremers HM, Doran MF, Turesson C, O'Fallon WM (2003). Survival in rheumatoid arthritis: a population-based analysis of
trends over 40 years. Arthritis Rheum.

[2] Tobon GJ, Youinou P, Saraux A (2010). The environment, geo-epidemiology, and autoimmune disease:
Rheumatoid arthritis. J Autoimmun.

[3] Rahman A, Isenberg DA (2008). Systemic lupus erythematosus. N Engl J Med.

[4] Yurkovich M, Vostretsova K, Chen W, Avina-Zubieta JA (2014). Overall and cause-specific mortality in patients with systemic
lupus erythematosus: a meta-analysis of observational
studies. Arthritis Care Res (Hoboken).

[5] Criswell LA, Pfeiffer KA, Lum RF, Gonzales B, Novitzke J, Kern M (2005). Analysis of families in the multiple autoimmune disease genetics
consortium (MADGC) collection: the PTPN22 620W allele associates with
multiple autoimmune phenotypes. Am J Hum Genet.

[6] Plenge RM, Seielstad M, Padyukov L, Lee AT, Remmers EF, Ding B (2007). TRAF1-C5 as a risk locus for rheumatoid arthritis--a genomewide
study. N Engl J Med.

[7] Tsao BP (2004). Update on human systemic lupus erythematosus
genetics. Curr Opin Rheumatol.

[8] Kalliolias GD, Ivashkiv LB (2016). TNF biology, pathogenic mechanisms and emerging therapeutic
strategies. Nat Rev Rheumatol.

[9] Dunham I, Sargent CA, Trowsdale J, Campbell RD (1987). Molecular mapping of the human major histocompatibility complex
by pulsed-field gel electrophoresis. Proc Natl Acad Sci USA.

[10] Choi SJ, Rho YH, Ji JD, Song GG, Lee YH (2006). Genome scan meta-analysis of rheumatoid arthritis. Rheumatology (Oxford).

[B11] Jahid M, Rehan-Ul-Haq, Jha PK, Chawla D, Avasthi R, Ahmed RS (2017). Tumor necrosis factor-α -308 polymorphism in North Indian
rheumatoid arthritis patients and association with mRNA and serum
*TNF-α*. Clin Rheumatol.

[12] Ramírez-Bello J, Cadena-Sandoval D, Mendoza-Rincón JF, Barbosa-Cobos RE, Sánchez-Muãoz F, Amezcua-Guerra LM, Sierra-Martínez M, Jiménez-Morales S (2018). Tumor necrosis factor gene polymorphisms are associated with
systemic lupus erythematosus susceptibility or lupus nephritis in Mexican
patients. Immunol Res.

[13] You CG, Li XJ, Li YM, Wang LP, Li FF, Guo XL (2013). Association analysis of single nucleotide polymorphisms of
proinflammatory cytokine and their receptors genes with rheumatoid arthritis
in northwest Chinese Han population. Cytokine.

[B14] Hussein YM, Mohamed RH, Pasha HF, El-Shahawy EE, Alzahrani SS (2011). Association of tumor necrosis factor alpha and its receptor
polymorphisms with rheumatoid arthritis in female patients. Cell Immunol.

[15] Rood MJ, van Krugten MV, Zanelli E, van der Linden MW, Keijsers V, Schreuder GM (2000). TNF-308A and HLA-DR3 alleles contribute independently to
susceptibility to systemic lupus erythematosus. Arthritis Rheum.

[16] Sun R, Huang Y, Zhang H, Liu R (2013). MMP-2, TNF-alpha and NLRP1 polymorphisms in Chinese patients with
ankylosing spondylitis and rheumatoid arthritis. Mol Biol Rep.

[17] Ates O, Hatemi G, Hamuryudan V, Topal-Sarikaya A (2008). Tumor necrosis factor-alpha and interleukin-10 gene promoter
polymorphisms in Turkish rheumatoid arthritis patients. Clin Rheumatol.

[18] Santos MJ, Carmona-Fernandes D, Caetano-Lopes J, Perpetuo IP, Vidal B, Capela S (2012). TNF promoter -308 G>A and LTA 252 A>G polymorphisms in
Portuguese patients with systemic lupus erythematosus. Rheumatol Int.

[19] Lee YH, Ji JD, Song GG (2007). Tumor necrosis factor-alpha promoter -308 A/G polymorphism and
rheumatoid arthritis susceptibility: a meta-analysis. J Rheumatol.

[20] Lee YH, Harley JB, Nath SK (2006). Meta-analysis of TNF-alpha promoter -308 A/G polymorphism and SLE
susceptibility. Eur J Hum Genet.

[21] Lv HZ, Lin Tao, Zhu XY, Zhang JT, Lu Jing (2011). Association analysis of single nucleotide polymorphism of
*TNF-α* and rheumatoid arthritis in northern Chinese Han
population [in chinese]. Chin J Cell Mol Immunol.

[22] Yen JH, Chen CJ, Tsai WC, Lin CH, Ou TT, Wu CC (2001). Tumor necrosis factor promoter polymorphisms in patients with
rheumatoid arthritis in Taiwan. J Rheumatol.

[23] McHugh NJ, Owen P, Cox B, Dunphy J, Welsh K (2006). MHC class II, tumour necrosis factor alpha, and lymphotoxin alpha
gene haplotype associations with serological subsets of systemic lupus
erythematosus. Ann Rheum Dis.

[24] Guo J, Jin M, Zhang M, Chen K (2012). A genetic variant in miR-196a2 increased digestive system cancer
risks: a meta-analysis of 15 case-control studies. PLoS One.

[25] Moher D, Liberati A, Tetzlaff J, Altman DG (2010). Preferred reporting items for systematic reviews and
meta-analyses: the PRISMA statement. Int J Surg.

[26] Higgins JP, Thompson SG (2002). Quantifying heterogeneity in a meta-analysis. Stat Med.

[27] Egger M, Davey Smith G, Schneider M, Minder C (1997). Bias in meta-analysis detected by a simple, graphical
test. BMJ.

[28] Fragoso JM, Vargas Alarcón G, Jiménez Morales S, Reyes Hernández OD, Ramírez Bello J (2014). Tumor necrosis factor alpha (*TNF-α*) in
autoimmune diseases (AIDs): molecular biology and genetics. Gac Med Mex.

[29] Chu WM (2013). Tumor necrosis factor. Cancer Lett.

[30] Khalilzadeh O, Noshad S, Rashidi A, Amirzargar A (2011). Graves' ophthalmopathy: a review of
immunogenetics. Curr Genomics.

[31] Feldmann M, Brennan FM, Maini RN (1996). Role of cytokines in rheumatoid arthritis. Annu Rev Immunol.

[32] Gomez D, Correa PA, Gomez LM, Cadena J, Molina JF, Anaya JM (2004). Th1/Th2 cytokines in patients with systemic lupus erythematosus:
is tumor necrosis factor alpha protective?. Semin Arthritis Rheum.

[33] Heward J, Gough SC (1997). Genetic susceptibility to the development of autoimmune
disease. Clin Sci (Lond).

[34] Becker KG, Simon RM, Bailey-Wilson JE, Freidlin B, Biddison WE, McFarland HF (1998). Clustering of non-major histocompatibility complex susceptibility
candidate loci in human autoimmune diseases. Proc Natl Acad Sci USA.

[35] Bayley JP, Ottenhoff TH, Verweij CL (2004). Is there a future for TNF promoter polymorphisms?. Genes Immun.

[36] Hajeer AH, Hutchinson IV (2001). Influence of TNFalpha gene polymorphisms on TNFalpha production
and disease. Hum Immunol.

[37] Allen RD (1999). Polymorphism of the human TNF-alpha promoter--random variation or
functional diversity?. Mol Immunol.

[38] Wilson AG, Symons JA, McDowell TL, McDevitt HO, Duff GW (1997). Effects of a polymorphism in the human tumor necrosis factor
alpha promoter on transcriptional activation. Proc Natl Acad Sci USA.

[39] Pociot F, D'Alfonso S, Compasso S, Scorza R, Richiardi PM (1995). Functional analysis of a new polymorphism in the human TNF alpha
gene promoter. Scand J Immunol.

[40] Kaijzel EL, van Krugten MV, Brinkman BM, Huizinga TW, van der Straaten T, Hazes JM (1998). Functional analysis of a human necrosis factor alpha (TNF-alpha)
promoter polymorphism related to joint damage in rheumatoid
arthritis. Mol Med.

